# Zinc deficiency correlates with severity of diabetic polyneuropathy

**DOI:** 10.1002/brb3.2349

**Published:** 2021-09-14

**Authors:** Mona Hussein, Wael Fathy, Amr Hassan, Rehab Abd Elkareem, Salma Marzouk, Yasmine Shawki Kamal

**Affiliations:** ^1^ Neurology Department Beni‐Suef University Beni‐Suef Egypt; ^2^ Department of Anaesthesia and Pain Management Beni‐Suef University Beni‐Suef Egypt; ^3^ Neurology Department Cairo University Cairo Egypt; ^4^ Department of Clinical and Chemical pathology Beni‐Suef University Beni‐Suef Egypt; ^5^ Neurophysiology Unit Neurology Department Cairo University Cairo Egypt; ^6^ Rashid Hospital Dubai United Arab Emirates

**Keywords:** diabetic polyneuropathy, MNSI score, NSC score, zinc

## Abstract

**Objectives:**

There are controversies about the role of zinc in the development of both types 1 and 2 diabetes. The aim of this study was to assess serum zinc level in diabetic patients with and without peripheral neuropathy in comparison to healthy controls and to explore the possible relationship between serum zinc level and severity of peripheral neuropathy.

**Methods:**

This case control study was conducted on 120 subjects: 40 patients fulfilled the criteria for diagnosis of probable diabetic polyneuropathy (DPN), 40 diabetic patients without polyneuropathy (N‐DPN) and 40 healthy controls. DPN patients were submitted to clinical assessment of diabetic neuropathy using neuropathy symptom and change (NSC) scale, Michigan Neuropathy Screening Instrument Physical Assessment (MNSI) scale and electrophysiological assessment using nerve conduction study. Zinc serum level was measured in all subjects included in this study using direct colorimetric test method.

**Results:**

Diabetic patients with and without neuropathy were found to have significantly lower mean values of serum zinc than healthy controls (*p* = .025,  .03 respectively). There is a statistically significant negative correlation between zinc serum level and hemoglobin A1C (HA1C) (*p* ˂ .001), NSC score (*p* = .001) and MNSI score (*p* = .003) in DPN group. There were also statistically significant correlations between zinc serum level and nerve conduction study values.

**Conclusion:**

Zinc deficiency significantly correlates with the severity of DPN and glycemic control.

## Graphical abstract

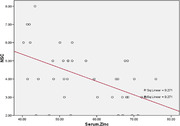

## 1 INTRODUCTION

Diabetes mellitus (DM) is a leading cause of morbidity and mortality all over the world. According to the International Diabetes Federation, by 2015, about 8.8% of adults aged 20–79 or 415 million people worldwide are estimated to have DM. By 2040, one adult in 10 or 642 million people will have DM (Allgot et al., 2015).

Diabetic peripheral neuropathy (DPN) affects approximately 30%–50% of all patients with DM (Deshpande et al., 2008). It can involve all peripheral nerves including sensory and motor neurons, and the autonomic nervous system as well. Signs and symptoms vary depending on the nerve fiber type affected. The proposed pathogenic mechanisms that contribute to DPN include microangiopathy and oxidative stress. Current clinical management guidelines for DPN are limited to symptomatic medical treatment for neuropathic pain and tight glycemic control (Dyck et al., 1993). Multiple clinical trials on patients with diabetic neuropathy revealed that oral supplementation with zinc helps in achieving better glycemic control and improvement in the severity of DPN as assessed by neurophysiological studies (Gupta et al., 1998; Hayee et al., 2005).

Zinc deficiency is thought to be one of the possible causes of the development of DM. Zinc is directly involved in the synthesis, storage and secretion of insulin (Chausme, 1998; Mocchegiani et al., 2008). It also maintains the structural stability of insulin. Furthermore, zinc deficiency was found to aggravate insulin resistance in noninsulin dependent DM (Zargar et al., 1998).

Zinc deficiency was also incriminated in the development of diabetic complications. This was attributed to its antioxidant effect and its contribution as a key component of many antioxidases. It inhibits the damage associated with lipid peroxidation and induces the clearance of free radicals (Thomas et al., 1986).

It has been reported that patients with diabetic neuropathy have a significant decrease in zinc serum level. This was explained by elevated lipid peroxidation in patients with zinc deficiency, which is one of the major causes for diabetic neuropathy (Migdalis et al., 2005).

The aim of this study was to assess serum zinc level in diabetic patients with and without peripheral neuropathy in comparison to healthy controls and to investigate the potential relationship between serum zinc level and severity of peripheral neuropathy.

## 2 SUBJECTS AND METHODS

### 2.1 Subjects

The present study is a cross‐sectional case‐control study that was conducted on 120 subjects: 40 patients with DPN, 40 diabetic patients without polyneuropathy (N‐DPN) and 40 age and sex matched healthy controls. Patients were recruited during the period between January 2019 and December 2019 from the Neurology and Pain Outpatient Clinics, Beni‐Suef University Hospital. Written informed consent was obtained from each participant in this study. The study was conducted in accordance with the Declaration of Helsinki and approved by local ethical committee, Faculty of Medicine, Beni‐Suef University.

We included diabetic patients aged 18–60 years from both sexes who fulfilled the American Diabetic Association criteria 2015 for diagnosis of DM disease which includes one of the following: fasting blood sugar (FBS) ≥126 mg/dl (7.0 mmol/L), 2 hours post prandial plasma glucose (2‐hour PG) ≥200 mg/dl (11.1 mmol/L), hemoglobin A1C (HbA1C) ≥6.5% (48 mmol/L) and random plasma glucose (PG) ≥200 mg/dl (11.1 mmol/L) in individuals with symptoms of hyperglycemia (American Diabetes Association, 2015). DPN patients recruited also were fulfilling the criteria for diagnosis of probable DN which include any two or more of the following: positive neuropathic sensory symptoms (e.g., “asleep numbness,” stabbing or prickling, aching or burning pain) predominantly in the toes, feet, or legs, diminished distal sensation or diminished or lost ankle reflexes (Tesfaye et al., 2010). We excluded patients with any concomitant medical or metabolic disorder known to cause neuropathy, e.g., thyroid or parathyroid disease, hepatic or renal impairment, autoimmune disorder, tuberculosis, patients with current or past history of malignancy, patients with a history of alcohol intake, substance abuse or regular intake of a drug known to cause neuropathy. Pregnant women were also excluded from the study.

### 2.2 Methods

Clinical and electrophysiological assessment of diabetic neuropathy were done only for diabetic patients with peripheral neuropathy, while laboratory assessment of zinc serum level was done for the three included groups.

- Clinical assessment of diabetic neuropathy:
- Neuropathy symptom and change (NSC) scale: It consists of questions regarding the type of neuropathic pain, time and location of symptoms, arousal from sleep and maneuvers relieving symptoms (NSC score of 3−4 = mild neuropathy, 5−6 = medium neuropathy and 7−9 = severe neuropathy) (Young et al., 1993).
- Michigan Neuropathy Screening Instrument Physical Assessment (MNSI) scale: It involves inspection of the feet for deformities, dry skin, hair or nail abnormalities, callous or infection, semi‐quantitative assessment of vibration sensation at the dorsum of the great toe, grading of ankle reflexes and monofilament testing. Patients were considered neuropathic when they scored >2 points (Moghtaderi et al., 2006).
- Laboratory assessment:
- HbA1C test: We measured HbA1C level for all diabetic patients included in this study. It was measured using a nephelometer by kits (Mispa‐i2; AGAPPE). This was applied specific protein analyzer (AGAPPE Diagnostic Switzerland GmbH). The normal range of HbA1C level is between 4.8% and 5.7%.
- Zinc serum level: It was measured for all patients and controls included in this study by kits (Reactivos GPL, Barcelona, Espana) using direct colorimetric test method. At pH 8.2 in a buffered media, zinc reacted with the specific complexant NITRO PAPS, forming a stable‐colored complex. The color intensity was proportional to the amount of zinc present in the sample. This was applied on semi‐automated photometer (ELITechGroup VITAL, Dieren ‐ The Netherlands). Note that 5 ml of whole blood was centrifuged for 10–15 min at 3000 rpm. Supernatant clear sera were collected and zinc was immediately measured at a wave length of 578 nm at 37°C. The zinc calibration value was verified using NIST (National Institute of Standards and Technology). The kit has detection limit of 4 μg/dl up to 1000 μg/dl. The reference range for normal serum zinc level is 68–107 μg/dl.
- Electrophysiological studies:
- Electrophysiological studies were done only for diabetic patients with peripheral neuropathy in the Neuro Diagnostic Research Center, Beni‐Suef University Hospital. Nerve conduction study record was done using Nihon Kohden Neuropack machine (Japan). Electrical stimulator and surfaces electrodes were used to assess Median and Ulnar nerves (motor and sensory parts) in upper limbs, Peroneal nerve (motor part), Tibial nerve (motor) and Sural nerve (sensory part) in lower limbs.

Statistical methods: The data were coded and entered using the statistical package for social science version 24 (SPSS v 24) (Released 2016. IBM SPSS Statistics for Windows, Version 24.0. IBM Corp, Armonk, NY, USA). Sample size calculation was conducted using G*Power version 3.1.9.2 Software, with a statistical power of 80% and a two‐sided 5% significant level. Descriptive statistics for diabetic patients and controls were reported as mean ± SD and number (%) for categorical variables. Independent sample student *t*‐test was used for comparison between diabetic patients with and without neuropathy in HA1C and disease duration. The one‐way analysis of variance (ANOVA) was used to assess statistical differences between the three study groups in age and serum zinc. Least significant difference (LSD) test was used for groups’ multiple comparisons in post hoc analysis. Chi square test was used for comparison between diabetic patients with and without neuropathy and controls in sex and antidiabetic drugs. The Pearson correlation coefficient (*r*) was used to describe the degree of relationship between serum level of zinc and HbA1C, scales of DPN and nerve conduction studies. The probability/significance value *p* ≥ .05 is not statistically significant and *p* < .05 is statistically significant.

## 3 RESULTS

The demographics of all participants and clinical characteristics of diabetic patients with and without neuropathy (disease duration, NSC score and MNSI score) and their mean HbA1C are shown in Table 1.

**TABLE 1:** Demographics and clinical characteristics of the study and control groups

		DPN (*n* = 40)	N‐DPN (*n* = 40)	Controls (*n* = 40)	*p*‐Value
Age (mean [SD])		51.5 (10.65)	55.05 (7.76)	51.53 (10.25)	.183
Sex	Male (n [%])	29 (72.5%)	25 (62.5%)	22 (55%)	.265
	Female (n [%])	11 (27.5%)	15 (37.5%)	18 (45%)	
Disease duration (mean [SD])		8.93 (6.39)	7.95 (4.68)	–	.439
Anti‐diabetic drugs	Oral hypo‐glycemics (n [%])	17 (42.5%)	20 (50%)	–	.501
	Insulin (n [%])	23 (57.5%)	20 (50%)	–	
NSC (mean [SD])		4.1 (1.55)			
MNSI (mean [SD])		4.73 (1.77)			
HbA1C in % (mean [SD])		7.99 (0.86)	7.56 (1.24)	–	.076

Abbreviations: DPN, diabetic polyneuropathy; N‐DPN, diabetic patients without neuropathy; NSC, neuropathy symptom and change; MNSI: Michigan Neuropathy Screening Instrument; HbA1C, hemoglobin A1C.

Results of nerve conduction study of median, ulnar, peroneal, sural and tibial nerves including distal latency, conduction velocity and amplitude are presented in Table 2.

**TABLE 2:** Results of nerve conduction studies in diabetic polyneuropathy (DPN) group

Examined nerve		DPN (*n* = 40) (mean [SD])
Median nerve (motor)	Conduction velocity	50.13 (7.75)
	Amplitude	7.22 (2.76)
	Latency	5.31 (1.56)
Median nerve (sensory)	Conduction velocity	39.07 (8.91)
	Amplitude	18.09 (10.86)
	Latency	4.74 (1.52)
Ulnar nerve (motor)	Conduction velocity	52.52 (7.23)
	Amplitude	8.04 (1.78)
	Latency	4.39 (1.47)
Ulnar nerve (sensory)	Conduction velocity	48.54 (7.7)
	Amplitude	17.11 (11.52)
	Latency	2.95 (0.58)
Peroneal nerve	Conduction velocity	42.4 (6.62)
	Amplitude	3.34 (1.7)
	Latency	6.83 (2.86)
Tibial nerve	Conduction velocity	37.98 (8.27)
	Amplitude	6.33 (3.24)
	Latency	6.99 (3.67)
Sural nerve	Conduction velocity	39.93 (8.73)
	Amplitude	5.8 (4.33)
	Latency	5.07 (1.94)

The mean serum zinc level in DPN group was significantly lower than healthy controls (56.95 ± 10.86, 62.09 ± 9.2 μg/dl, respectively) (*p* = .023). The mean serum zinc level in N‐DPN group was significantly lower than healthy control (57.198 ± 9.83, 62.09 ± 9.2 μg/dl, respectively) (*p* = .03). No statistically significant difference was found between serum zinc level in DPN and N‐DPN groups (*p* = .911) (Table 3).

**TABLE 3:** Serum zinc level among study and control groups

				*p*‐Value			
	DPN (*n* = 40)	N‐DPN (*n* = 40)	Controls (*n* = 40)	Total *p*‐value	DPN versus N‐DPN	DPN versus control	N‐DPN versus control
Serum zinc (μg/dl)	56.95 (10.86)	57.198 (9.83)	62.09 (9.2)	.038*	.911	.023*	.03*

**^Abbreviations:^ :**DPN, diabetic polyneuropathy; N‐DPN, diabetic patients without neuropathy.

* *p*‐Value ≤ 0.05 (significant).

**FIGURE 1:**
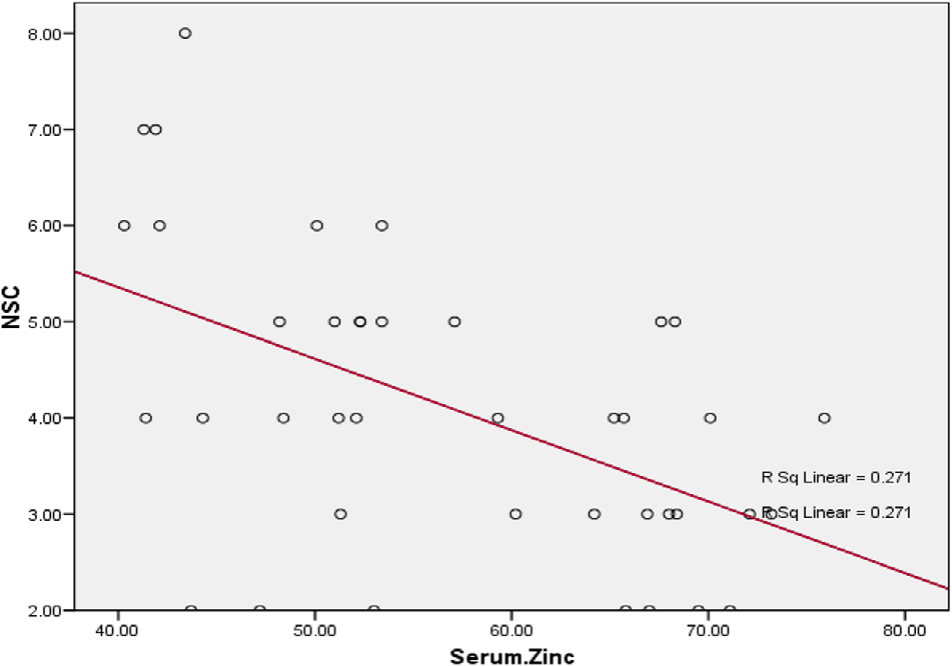
Correlation between serum zinc level and neuropathy symptom and change (NSC) scores

**FIGURE 2:**
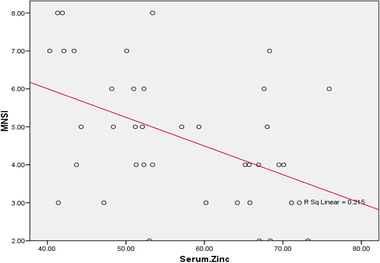
Correlation between serum zinc level and Michigan Neuropathy Screening Instrument scores (MNSI) scores

There was a statistically significant negative correlation between zinc serum level in DPN group and HbA1C (*p* ˂ .001), NSC score (*p* = .001) (Figure 1) and MNSI score (*p* = .003) (Figure 2).

There was a statistically significant positive correlation between zinc serum level and conduction velocity of median nerve (motor branch), ulnar nerve (motor branch), peroneal nerve and sural nerve in diabetic patients with peripheral neuropathy. Zinc serum level in those patients was also positively correlated with amplitude of median nerve (motor branch), ulnar nerve (motor and sensory branch), peroneal nerve, tibial nerve and sural nerve. There was a statistically significant negative correlation between zinc serum level and distal latency of median nerve (motor branch) and sural nerve (Table 4).

**TABLE 4:** Correlation between serum zinc level and the values of nerve conduction study among diabetic polyneuropathy (DPN) group

		Serum zinc in μg/dl	
Values of nerve conduction study		coefficient (*r*)	*p*‐Value
Median nerve (motor)	Conduction velocity	0.738	<.001^*^
	Amplitude	0.499	.001^*^
	Latency	−0.323	.042^*^
Median nerve (sensory)	Conduction velocity	0.168	.3
	Amplitude	0.154	.341
	Latency	−0.106	.513
Ulnar nerve (motor)	Conduction velocity	0.398	.011^*^
	Amplitude	0.528	<.001^*^
	Latency	−0.157	.334
Ulnar nerve (sensory)	Conduction velocity	0.192	.235
	Amplitude	0.398	.011^*^
	Latency	−0.085	.602
Peroneal nerve	Conduction velocity	0.483	.002^*^
	Amplitude	0.609	<.001^*^
	Latency	0.15	.354
Tibial nerve	Conduction velocity	0.277	.083
	Amplitude	0.534	<.001^*^
	Latency	−0.126	.438
Sural nerve	Conduction velocity	0.495	.001^*^
	Amplitude	0.369	.019^*^
	Latency	−0.395	.012^*^

## 4 DISCUSSION

It is evident from the literature that zinc plays an important role in β‐cell function, insulin action, glucose homeostasis and consequently affecting pathogenesis of DM. Additionally, zinc deficiency can be considered one of the possible causes for the development of diabetic complications because of its antioxidant properties. Zinc is a key component in many antioxidant enzymes (Chausme, 1998).

Our results revealed that diabetic patients with and without neuropathy have significantly lower serum zinc level than healthy control group. There was a statistically significant negative correlation between zinc serum level and HbA1C in patients with diabetic neuropathy.

In accordance with our findings, Rai et al. (1997) showed that serum zinc concentration was lower in diabetics when compared to healthy controls and found a negative correlation between serum zinc and glycated proteins. Similarly, other several studies reported that serum zinc was significantly lower in diabetic patients compared to healthy controls (Al‐Maroof & Al‐Sharbatti, 2006; Masood et al., 2009; Nsonwu et al., 2009; Santa et al., 2014; Viktorínová et al., 2009).

The reported zinc deficiency in diabetic patients in different studies was explained by multiple theories. Zinc plays an important role in glucose and lipid metabolism. It decreases glucose absorption and synthesis, while promotes glucose metabolism and storage. This is primarily achieved via enhancing the activity of the key enzymes involved in these metabolic processes, such as phosphokinase, α‐glucosidase and glycogen synthase. Zinc also plays an important role in the normal functioning of the islet cells of the pancreas as it mediates insulin production and efficient packaging into vesicles (Rungby, 2010). Additionally, zinc was found to increase insulin sensitivity by increasing the binding ability of insulin to its receptors (Chausme, 1998).

However, our results were contradictory with the findings of other studies that reported a significant increase in serum zinc in diabetic patients compared to controls (D'Ocon et al., 1987; Mamza et al., 2016; Mateo et al., 1978; Osman et al., 2004). Additionally, Akhuemokhan et al. (2010) showed that serum concentration of zinc had no significant correlation with either FBS or HbA1C. Moreover, some studies considered zinc deficiency a consequence and not a cause of DM due to increased urinary loss and/or decreased gastrointestinal absorption of zinc (Jaswant & Tajinder, 2015). In another study, levels of zinc in diabetic patients were found to be equal to or higher than that of control groups. Interestingly, they attributed the presence of vascular complications in DM to higher level of serum zinc concentration (Rusu et al., 2005).

A systematic review and meta‐analysis on 25 studies that investigated the effects of zinc supplementation on clinical and biochemical parameters in patients with diabetes demonstrated that zinc supplementation had beneficial effects on glycemic control and lipid profile (Jayawardena et al., 2012).

Our results revealed a statistically significant negative correlation between zinc serum level in diabetic patients and both MNSI and NSC scores. There were also statistically significant correlations between zinc serum level and nerve conduction study values indicating increased severity of diabetic neuropathy in patients with lower serum level of zinc.

In accordance with our findings, Hayee et al. (2005) showed that serum zinc levels at baseline were significantly lower in patients with diabetic neuropathy when compared with healthy controls. Similarly, another study revealed that patients with diabetic neuropathy had significantly lower serum zinc levels at baseline than healthy controls. After 6 weeks of zinc therapy, there was significant improvement in values of FBS, 2‐hour PG and conduction velocity in median and common peroneal nerve (Gupta et al., 1998). Zinc had been reported to have a protective effect against diabetes‐induced peripheral nerve damage by promoting synthesis of metallothionein and downregulating oxidative stress in diabetic rats (Liu et al., 2014).

The beneficial effect of oral zinc supplementation on diabetic neuropathy can be explained by the antioxidant properties of zinc (Singh et al., 1998). Zinc is a component of the important antioxidant enzyme superoxide dismutase (Cu‐Zn SOD) (Tolonen, 1990). Additionally, zinc has a role in the release of vitamin A from its storage site in the liver (Goode et al., 1991; Oberley, 1988).

The correlation between serum zinc and the severity of diabetic neuropathy can be explained by the evident association between zinc deficiency and poor glycemic control which is known to aggravate symptoms of diabetic neuropathy. Furthermore, patients with zinc deficiency are less protected against oxidative stress induced peripheral nerve damage than patients with normal zinc.

The main conclusion of this study is that zinc deficiency significantly correlates with the severity of DPN and glycemic control. Moreover, diabetic patients with and without neuropathy have significantly lower zinc level than healthy controls.

Further studies should be conducted in those with prediabetes and diabetic patients without peripheral neuropathy to evaluate potential beneficial effects of zinc supplementation in prevention of diabetic neuropathy after taking into consideration the risk of copper‐deficiency myeloneuropathy in patients with zinc excess.

## CONFLICT OF INTEREST

The authors declare no conflict of interest

## AUTHOR CONTRIBUTIONS

Mona Hussein participated in study design, neurophysiological studies and helped to draft the manuscript. Wael Fathy participated in study design, collection and analysis of data and helped to the draft manuscript. Rehab Abd Elkareem participated in study design, performed the laboratory work and helped to the draft manuscript. Amr Hassan and Yasmine Shawki Kamal revised the manuscript. All authors read and approved the final manuscript with agreement to be accountable for all aspects of the work in ensuring that questions related to the accuracy or integrity of any part of the work are appropriately investigated and resolved.

## Data Availability

The datasets used and/or analyzed during the current study are available from the corresponding author on reasonable request with permission of Faculty of Medicine, Beni‐Suef University, Egypt.
